# A brief historicity of the *Diagnostic and Statistical Manual of Mental Disorders*: Issues and implications for the future of psychiatric canon and practice

**DOI:** 10.1186/1747-5341-7-2

**Published:** 2012-01-13

**Authors:** Shadia Kawa, James Giordano

**Affiliations:** 1 Basic Biobehavioral and Psychological Sciences Branch, Behavioral Research Program, Division of Cancer Control and Population Sciences, National Cancer Institute, 6130 Executive Blvd., EPN 4066, Bethesda, MD 20892, USA; 2 Center for Neurotechnology Studies, Potomac Institute for Policy Studies, 901 N. Stuart St. Suite 900, Arlington, VA 22203, USA; 3 Oxford Centre for Neuroethics, Uehiro Centre for Practical Philosophy, University of Oxford, OX1, 1PT Oxford, UK

**Keywords:** Psychiatry, *DSM*, classification, nosology, psychopathology, mental disorders, historicity

## Abstract

The Diagnostic and Statistical Manual (*DSM*) of the American Psychiatric Association, currently in its fourth edition and considered *the *reference for the characterization and diagnosis of mental disorders, has undergone various developments since its inception in the mid-twentieth century. With the fifth edition of the *DSM *presently in field trials for release in 2013, there is renewed discussion and debate over the extent of its relative successes - and shortcomings - at iteratively incorporating scientific evidence on the often ambiguous nature and etiology of mental illness. Given the power that the *DSM *has exerted both within psychiatry and society at large, this essay seeks to analyze variations in content and context of various editions of the *DSM*, address contributory influences and repercussion of such variations on the evolving landscape of psychiatry as discipline and practice over the past sixty years. Specifically, we document major modifications in the definition, characterization, and classification of mental disorders throughout successive editions of the *DSM*, in light of shifting trends in the conceptualization of psychopathology within evolving schools of thought in psychiatry, and in the context of progress in behavioral and psychopharmacological therapeutics over time. We touch upon the social, political, and financial environments in which these changes took places, address the significance of these changes with respect to the legitimacy (and legitimization) of what constitutes mental illness and health, and examine the impact and implications of these changes on psychiatric practice, research, and teaching. We argue that problematic issues in psychiatry, arguably reflecting the large-scale adoption of the *DSM*, may be linked to difficulties in formulating a standardized nosology of psychopathology. In this light, we highlight 1) issues relating to attempts to align the *DSM *with the medical model, with regard to increasing specificity in the characterization of discrete mental disease entities and the incorporation of neurogenetic, neurochemical and neuroimaging data in its nosological framework; 2) controversies surrounding the medicalization of cognition, emotion, and behavior, and the interpretation of subjective variables as 'normal' or 'abnormal' in the context of society and culture; and 3) what constitutes treatment, enablement, or enhancement - and what metrics, guidelines, and policies may need to be established to clarify such criteria.

## Introduction

Translated into over twenty languages, referred to by clinicians from multiple schools, as well as by researchers, policy-makers, criminal courts, and third-party reimbursement entities [1], the *Diagnostic and Statistical Manual *of the American Psychiatric Association (*DSM) *enjoys a nearly hegemonic status as *the *reference for the assessment and categorization of mental disorders of all types - not only in the United States, but increasingly in Europe and more recently Asia. To be sure, the discipline and practice of psychiatry has changed since the first *DSM *was released, and with the fifth edition (*DSM-V*) currently in field trials in preparation for general release in spring 2013, there is renewed discussion - and debate - about whether this latest volume represents 1) a work of lessons learned from prior editions, and in this way may be seen as an epistemologically iterative step in defining and characterizing the often ambiguous if not enigmatic qualities of "mental disorder(s)", or 2) merely an embellished version of previous volumes that perpetuates misnomers and vagaries and thus fails to be anything more than of nominal value.

Whether endorsed as a reasonable gold-standard or criticized as limited in scope and utility, what cannot be ignored is the effect - if not power - that the *DSM *has exerted, and continues to exert, both within psychiatry and society at-large. Therefore, it is important to consider if, and how the *DSM-V *will manifest impact in and upon the character and conduct of psychiatry, medicine and the social sphere. Toward this end, we pose a Socratic question - from where have the *DSM *and psychiatry come, and to where are they going? Thus, this essay seeks to analytically scrutinize - and contextualize - the major developments that have occurred in various editions of the *DSM*, focusing upon factors that motivated its development in 1952, and the multiple changes and repercussions various editions have effected in psychiatry over the past sixty years. Inquiry to the history of such a prominent standardized nosology of mental disorders may be a means of probing (at least in contours and highlights) the intellectual landscape of psychiatry throughout the second half of the twentieth century. In this light, three major "phases" will be addressed: first is the period encompassing the formulation and release of the first and subsequent second edition of the *DSM; *second is the period of the rather 'revolutionary' *DSM-III*, and third is the post-*DSM-III *period to the present, during which the *DSM-IV *and *DSM-IV Text-Revised (DSM-IVTR) *editions were released. Particular emphasis shall be upon the development of the *DSM-III*, as the major trends it embodied have been largely accentuated throughout all subsequent editions of the manual.

However, it may first be worth noting one of the most pervasive, yet often elusive characteristics of any historiological recollection, including this one. Namely, that by the very selection of key historical phenomena deemed to be worthy of inclusion in any account, and by the differential emphasis on, and chronological linkages between the diverse components of such an account, the final product is often far from being wholly "objective" and\or comprehensive. We recognize this, and acknowledge that such implicit tendencies may indeed have affected the scope and tenor of the present essay. Still, we hope to shed light on critical developments within and around the *DSM *throughout its history, and to attempt to make sense of this progression through a didactic approach. We shall also attempt to avoid cursory generalizations, by instead seeking a leveled appraisal of multiple, if at times conflicting, interpretations of the same historical phenomena.

### A beginning: Nosological attempts culminating in the *DSM-I*

Some of the first attempts to formally classify psychopathology in the United States were undertaken during the early nineteenth century. For the most part, such efforts were collections of demographic data by the Bureau of the Census, and were intended more for mental health policy to regulate the treatment of the institutionalized mentally ill, rather than for diagnostic purposes [2]. Uncertainty surrounding the etiological bases of psychopathology, and psychiatrists' contention that abnormal behavior involved complex, variable, and often obscure interactions of internal and external factors, compelled formulation of a uniform nosological system of acknowledged clinical utility (notable exceptions to such general uncertainties include the late nineteenth century association between syphilis and general paresis, and the early twentieth century identification of the link between vitamin B deficiency and pellagra) [3].

As the perceived role of psychiatry broadened to include mental health beyond the boundaries of mental institutions, interest in devising a viable classification system for psychopathological conditions was strengthened within the psychiatric community. In 1918, under the auspices of the Bureau of the Census and the National Committee for Mental Hygiene, the American Medico-Psychological Association (now the American Psychiatric Association), first attempted the creation of a formal, standardized nomenclature of psychopathological conditions. These efforts culminated in the publication of the *Statistical Manual for the Use of Institutions for the Insane*, what can be considered to be the predecessor of the *Diagnostic and Statistical Manual of Mental Disorders *series. The volume included 22 diagnostic categories, most of which were psychotic conditions associated with presumed somatic etiology. This biologically-oriented classification approach, consistent with then-dominant Kraepelinian constructs linking abnormal behavior to organic brain dysfunctions, echoed the nature of the psychiatric profession at the time: the overwhelming majority of clinicians practiced in mental asylums, where patient populations were predominantly afflicted with severe mental disturbances associated with conspicuous physical impairments and disease [2].

Nine subsequent editions of the *Statistical Manual *followed during the interwar years, all of which emphasized the somatic viewpoint, offered relatively broad categorizations of mental disorders, and were of limited diagnostic utility. The psychodynamic approach (mostly reflecting the work of Freud in Europe, and Meyer in the United States), which originated at the beginning of the twentieth century but had maintained an initially marginal role, ascended in dominance after the relative successes of this approach in treating military patients who suffered neuropsychiatric trauma [2]. Growing recognition of the role environmental stressors in mild psychological disturbances - "psychoneuroses" in the verbiage of the psychodynamic tradition - expanded psychiatrists' role in treating the milder emotional and behavioral disorders that were relatively common in society at-large. The conceptualization of psychopathology had largely shifted from recognizing mental conditions as discrete disease entities distinct from mental health, to considering mental health and illness on a continuum of variable severity [4].

Psychodynamic theory gained rapid acceptance in both the clinical and academic arenas of psychiatry, and by 1946, was officially acknowledged as the leading school of thought by the American Board of Psychiatry [2]. Respective of changing conceptualizations of mental disease, and a broadening of psychiatric clientele - both of which being for the most part incompatible with earlier nosological frameworks, the APA Committee on Nomenclature and Statistics sought to create a new classification system: the first edition of the *Diagnostic and Statistical Manual of Mental Disorders (DSM-I)*, which was officially released in 1952 [5]. This compendium included 102 broadly-construed diagnostic categories that were based upon psychodynamic etiological explanations, and were accordingly subdivided into two major groups of mental disorders: 1) conditions assumedly caused by organic brain dysfunction (associated with somatic disturbances such as intoxication, trauma, or a variety of physiological diseases), and 2) conditions presumed to result from the effect(s) of socio-environmental stressors on individuals' biological constitution and patients' inability to adapt to such pressures [6]. The latter group was further subdivided into psychoses - that is, those disorders constituting relatively severe conditions such as manic-depressive disorder or schizophrenia, and, at the other end of the scale, psychoneuroses, which included conditions such as anxiety, depressive disorders, and personality disorders [6]. A notable figure in the development and promotion of the psychodynamically-oriented "maladjustment model" fostered by the *DSM-I *was Adolf Meyer, a neurologist by training, whose interests shifted to psychiatry upon his move from Switzerland to the United States in 1892 [7,8]. While hailed as meaningful by its authors, the *DSM-I *actually had only limited bearing on psychiatric practice [9], although it did in fact set the stage for increasingly standardized categorization(s) of mental disorders, if not an implicit standardization of psychiatric approaches to diagnosis and treatment.

To compensate for perceived inadequacies of the *DSM-I*, the second edition of the *Diagnostic and Statistical Manual of Mental Disorders *was published in 1968, and was still largely reflective of the psychodynamic tradition [10], although this school of thought was already on the decline by the end of the 1960s, and subtle amendments made to *DSM-II *hinted (albeit somewhat inconspicuously) at such change. Two major trends can be noted in the content modifications to the *DSM-II*. The first was a further expansion of the definitions of mental illness that was arguably in line with a broadening of psychodynamic theory to be more inclusive of milder conditions seen in the general population. This was indicated by the addition of diagnostic categories such as "Conditions Without Manifest Psychiatric Disorder" for "... individuals who are psychiatrically normal but who nevertheless warrant examination by a psychiatrist", and "Transient Situational Disturbances" for "... disturbances of psychotic proportion ... when they are considered clearly transient reactions to overwhelming environmental stress" [11]. The second trend was an increased systematic categorization and specificity that suggested a return to the Kraepelinian tradition. This was evidenced by multiple subdivisions of former disorder categories, such as the addition of eight new "alchoholic brain syndromes", an increased number of "qualifiers" from four in the *DSM-I *to nine in the *DSM-II *- namely, "acute; chronic; not psychotic; mild; moderate; severe; in remission", and the explicit advocacy that clinicians "... diagnose every disorder that is present, even if one is the symptomatic expression of another" [11]. Yet another alteration in the *DSM-II *was the removal of the psychodynamic term "reaction", referring to the maladaptive response of an individual to socio-environmental sources of distress. A disclaimer accompanied the announcement of such modification:

"Some individuals may interpret this change as a return to a Kraepelinian way of thinking, which views mental disorders as fixed disease entities. Actually this was not the intent of the APA Committee on Nomenclature and Statistics: "[The Committee] tried to avoid terms which carry with them implications regarding either the nature of a disorder or its causes [...]. In the case of diagnostic categories about which there is current controversy concerning the disorder's nature or cause, the Committee has attempted to select terms which it thought would least bind the judgment of the user." [11]

While significant discrepancies existed between the classification schemes of the *DSM-I *and the *International Classification of Diseases, 6^th ^revision (ICD-6) *of the World Health Organization (WHO), The *DSM-II *And the *ICD-8 *were more closely aligned, reflecting a collaborative effort between the WHO and American psychiatrists sent to Europe prior to the publication of both the *ICD *and *DSM *manuals that same year [11]. It is interesting that Spitzer was an influential consultant to the *DSM-II*, a primary author of the "revolutionary" third edition of the *DSM*, and chaired the task force overseeing *DSM-III *development [12].

### A "turning point" in psychiatry: The *DSM-III*

Negative critique of psychiatry mounted considerably during the 1960s and early to mid 1970s; perhaps most notable was Thomas Szasz's 1961 challenge to the fundamental premise that all psychiatric conditions were "true illnesses", which by extension, cast skepticism upon the legitimacy of psychiatry as a medical discipline [12]. Moreover, the lack of clear demarcations between mental health and illness, and the relatively low reliability of psychiatric diagnoses, were sharply criticized both within the psychiatric community and from without [13]. In addition to the rising discontent towards psychodynamic psychiatry was a significant restriction of resource funding. Diminished research support from the National Institute of Mental Health (NIMH), and reduced resource allocations by the federal government and insurance providers were prevalent throughout the 1970s, based, at least in part, upon the perception that psychodynamic psychiatry's lack of uniformity in classification fostered non-rigorous investigations, and superfluous healthcare expenditures [12,14]. In particular, psychiatrists' non- medically licensed competitors (e.g.- psychologists, social workers, and counselors) were offering psychodynamically-based therapeutic services at significantly less expensive rates, and this challenged the psychiatric community to prove that its diagnoses and therapies were (more) efficacious and represented treatment of legitimate medical *diseases *[14]. Concomitantly, a neo-Kraepelinian "invisible college" of more biologically-oriented psychiatrists increasingly and more ardently criticized psychodynamically-oriented psychiatric research and practice [13].

Substantive advances in psychometric instruments for quantitative psychiatric assessment, such as rating scales and checklists for anxiety and depression, had become something of a standard in mental health research and practice. Progress in therapeutics had also ensued, with increasingly more efficient behavioral and brief psychotherapeutic approaches [13], and notably, progress in psychopharmacology, which by the 1960s had developed a significant armamentarium of mood- and behavior-altering agents [15]. In fact, by the mid 1970s, prescribing psychotropic medications had become *de rigueur *for much of psychiatric practice [16]. Posturing against the challenges facing psychiatry, the research community engaged series of responses; among the most significant being 1) a 1965 conference addressing psychiatric classification that was sponsored by the Psychopharmacology Research Branch of the NIMH, 2) the formulation of the Washington University criteria for operational diagnosis in the early 1970s, and 3) the development of the Research Diagnostic Criteria (RDC) by the NIMH Psychobiology of Depression Collaborative Study, in 1978 [17]. Thus, a multitude of factors created a propitious climate for change that culminated in the publication of the third edition of the *Diagnostic and Statistical Manual of Mental Disorders *in 1980 [18].

Many of the modifications incorporated into the *DSM-III *constituted a veritable paradigm shift in psychiatry. Perhaps the most telling feature of this trend was the official removal of the psychodynamic term "neurosis". Confronted by an outcry from psychodynamically-oriented psychiatrists, the decision to ban the term from the *DSM *was only mildly amended: the *DSM-III*'s Nomenclature Task Force eventually decided to include the term parenthetically as "neurotic disorder" after renaming mental disorder categories that corresponded to the earlier "neurosis" classifications. Additionally, an explanation of the distinction between "neurosis" as an etiological explanation, and the expression "neurotic disorder" as a discrete entity was underscored in the Introduction of the *DSM-III *[19]. This decision involved a categorical reorganization of many of the disorders previously conceptualized in psychodynamic terminology.

The increased number of mental disorder categories (from 182 in the *DSM-II *to 265 in the *DSM-III*) was posed to reflect the increase in psychiatric knowledge accrued since the *DSM-II*, as well as amplification in the *specificity *of and in diagnosis sought by the *DSM-III *Task Force. The latter often entailed ramifications from previous, more broadly construed categories into several individual "subtypes", each considered as a separate and discrete mental disorder. For instance, the number of disorders under the umbra of Schizophrenia changed from 14 categories in the *DSM-II *to 18 in the *DSM-III*. Several single disorders were divided into a number of distinct categories; for example, the *DSM-II *category "Specific Learning Disturbance" was divided into five different "Specific Developmental Disorders", the category "Tic" gave way to three distinct "Stereotyped Movement Disorders," and the single category "Feeding Disturbance" was replaced by four specific "Eating Disorders". This trend of subdivision and reclassification was most pronounced, however, for the categories previously classified as "neuroses". For instance, three classes of "Hysterical Neurosis" in the *DSM-II *gave way to six renamed "daughter disorders" in the *DSM-III*. The single category "Phobic Neurosis" was divided into five classes of "Phobic *Disorders*", and the single category "Depressive Neurosis" was substituted by four categories of "Major Depression" [20].

Additionally, many novel disorder categories (that were absent in the *DSM-II*) were formalized as mental illness in the *DSM-III*. These included Post-Traumatic Stress Disorder, an array of childhood and adolescence disorders (such as three categories of Attention-Deficit Disorder), seven classes of "Psychosexual Dysfunctions", and four "Disorders of Impulse Control Not Elsewhere Classified" (such as "Pathological Gambling"). Controversy has arisen over the legitimacy of several of these categories as "true illnesses" since their incorporation in the *DSM-III *and perpetuation in successive editions of the *DSM-IV*.

One of the amendments that was of greatest impact was the permanent removal of the category "Homosexuality" from the *DSM-II*. The change was originally made upon the publication of the seventh printing of the *DSM-II *in 1973, following a vote by the American Psychiatric Association earlier that year [14]. The decision was provocative (if not controversial) within camps of both advocacy and antagonism: in the former because it was contended (and in retrospect rightly so) that the earlier classifications of homosexuality-as-disorder were largely shaped by politically and socio-culturally contingent notions of deviance, rather than scientific corroboration [13], and in the latter because it was claimed that the change was based on consensus between figures representing "expert opinion" and Gay Rights' lobbying efforts.

In addition to content modifications, several other novel features of the *DSM-III *merit mention. Specifically, elaborate and more explicitly defined operational criteria for inclusion and exclusion were formulated for each disorder. These included standards for differential diagnosis of several categories of disorder that share similar characteristics, and the minimum duration of signs and symptoms required for a clinical diagnosis to be made. Another unique feature of the *DSM-III *was the adoption of a "multi-axial system" of diagnosis to account for patients' multi-factorial presentation and multi-dimensional experience of mental illness, and to facilitate a more comprehensive depiction of the patient's condition [21]. Finally, prior to adoption, the diagnostic categories developed in the *DSM-III *were subjected to NIMH-sponsored field trials between September 1977 and September 1979 in order to assess inter-rater reliability. While not compared to inter-rater agreement(s) on earlier nosologies, the *DSM-III *classification system was reported to have relatively good diagnostic reliability [11,22].

Taken together, the multiple amendments introduced to the *DSM-III *demonstrate a shift in the conceptualization of mental disorders from psychological "states" to discrete, operationally defined disease categories, and a return to a descriptive, symptom-based classification. In essence, the *DSM-III *inaugurated an attempt to "re-medicalize" American psychiatry.

### Repercussions

The release of the *DSM-III *in 1980 has been perceived as a nothing short of a momentous phenomenon. The APA's executive officer Melvin Sabshin heralded it as the victory of "science over ideology" [23], and Gerald Klerman, a leading psychiatrist at the time of the *DSM-III*'s inception, termed its development a "fateful turning point in the history of the American psychiatric profession" [24]. Effects of the *DSM-III *rapidly pervaded psychiatric practice, research, and teaching. As early as 1982, Klerman asserted that "... there is not a textbook of psychology or psychiatry that does not use *DSM-III *as the organizing principle for its table of contents and for classification of psychopathology". In fact, as Young has stated, "American medical schools and residency programs routinely expected students and physicians to pass examinations based on *DSM-III *criteria. [...] Both referees and journal editors expected manuscripts submitted to scholarly journals to be written in its language, and it was simply assumed that psychiatric research proposals would conform to its conventions" [25].

In an appraisal of the *DSM-III *a mere six years after its publication, Klerman noted a number of repercussions within several schools of thought in psychiatric research and clinical practice. Klerman claimed that the *DSM-III *had provided a formal common language that facilitated communication between multiple mental health professionals. While contending that the *DSM-III *had not become "the final consensus" with which to unify divergent perspectives regarding psychopathology, he acknowledged the "increasing acceptance of this diagnostic framework as the basis for teaching and research" [13]. Even proponents of the psychodynamic tradition, some of *DSM-II*'s major critics, gained a greater appreciation for the manual's classification system, while hoping for the addition of another "Axis" that would be more aligned with psychodynamic theory in the next edition of the *DSM-III *(an aspiration which, while explicitly suggested to the *DSM-III*'s Task Force and tentatively agreed upon by Spitzer and colleagues, never actually materialized) [13]. Indeed, while a torrent of criticism met the publication of the *DSM-III*, the "revolution" it fostered was quick and its effects durable, and psychiatrists who wished to retain their roles and credibility in the field soon had to conform to its newly introduced, government-sanctioned nosology of mental disorders [14].

The delineation of operationally defined diagnostic categories for mental disorders incurred a surge in epidemiological morbidity studies. Perhaps the most significant was the NIMH Epidemiological Catchment Area Project, which sought to assess the incidence and prevalence of mental disorders, as classified by the *DSM-III*, within the United States' general population [26]. Furthermore, while the *DSM-III *classification system did not explicitly link diagnostic categories to any particular treatment options, the symptom-based, somatically-oriented nature of the classification scheme was particularly compatible with biological therapies customized to discretely constructed disease entities. For any medication to be approved by the FDA, a drug needs to be proven effective in the treatment of a specific disease [14]. The clear demarcation of standardized, purportedly more reliable psychopathological diagnostic categories thus provided researchers, and pharmaceutical companies, an incentive to launch randomized controlled trials (RCT) to test newly developed psychopharmacological agents in the treatment of specific *DSM-III *disorders [14]. In the years following the publication of the *DSM-III*, billions of dollars were allocated by the government and pharmaceutical companies for psychopharmacological research [27]. For example, during the 1980s, the federal research budget allocated to the NIMH increased by 84 percent, to about $484 million annually [28]. Insurance providers equally welcomed the arrival of the new nosology, and adopted the *DSM-III *(and its subsequent editions) as the standard diagnostic categorization upon which to base reimbursement of therapeutic modalities (particularly, psychopharmacologic interventions) [14].

### After *DSM-III*: Accentuation of the "revolutionary textbook's" orientation?

Subsequent editions of the *Diagnostic and Statistical Manual of Mental Disorders *all displayed characteristics that are congruent with the orientation of the *DSM-III*. The number of categories of disorders has increased from 265 disorders in the *DSM-III*, to 292 in the *DSM-III-R*, to 297 in the *DSM-IV *[6,10,18,29-31]. The trend toward enhanced specificity of operational criteria has likewise become more pronounced throughout successive editions of the *DSM*, and information regarding prevalence, age- and sex -differential characteristics, and co-morbidity with other disorders has been added and regularly updated since the *DSM-III-R*. The significant increase in epidemiological studies based upon *DSM *criteria following the publication of the *DSM-III *has allowed for the incorporation of empirical data into the classification of several disorders [18,29-32]. Additions to the *DSM*s have also included information gathered from studies of the pathophysiology of mental disorders, and most recently have included data obtained from and based upon neuroimaging studies. The trend toward increased subdivisions of disorders that was originally initiated in the *DSM-III *has been evident in all subsequent editions of the manual. For instance, the eight categories of "Psychosexual Dysfunctions" in the *DSM-III *evolved to ten categories of "Sexual Dysfunctions" in the *DSM-III-R*, and to seventeen different "Sexual Dysfunction" classes in the *DSM-IV *and its text revised edition, the *DSM-IV-TR *[18,29-31]. Other major amendments included the incorporation of a section dealing with "Culture-bound Syndromes" in the *DSM-IV *and *DSM-IV-TR*, thereby acknowledging cultural variability in the ways that mental health and illness are expressed and construed [30,31]. In all, the changes to editions of the *DSM *following the *DSM-III *have essentially revolved around an accentuation of a "medicalizing" trend in psychiatry.

Rogler has attempted to interpret the increasing size and complexity of the *DSM *since its first edition until the *DSM-IV*, and has identified five major changes in its evolution; these are: 1) a theoretical shift in the conceptualization of mental disorders from a bio-psychosocial model to a research-oriented, medical model; 2) development of the multi-axial diagnostic system that facilitated a rise in biomedical findings based upon the five axes and the relation(s) between them; 3) the inclusion of new disorders and expansion of previously defined disorders; 4) a "lateral" reorganization of disorders into discrete, broad categories that entailed merging a number of disorders and eliminating others; and 5) a neo-Kraepelinian paradigm shift that was first evidenced in the *DSM-III*, that reinforced the descriptive, somatic orientation that then became the norm in all subsequent *DSM*s [33]. Indeed, this last point is important, as Rogler's analysis highlighted the iterative dominance of the medical model since the *DSM-III *- and, in parallel, the growth of this model in throughout almost all of psychiatric practice, education, and training.

At present, field trials of the *DSM-V *are underway, and the objectives outlined in "*A Research Agenda for DSM-V*" (2002) are largely concordant with the trends observed throughout the manual's evolution. Of particular interest is the continuing emphasis on the (at least implicit) incorporation of biological data into the classification of disorders in the *DSM*, with the intended elaboration of findings from studies in behavioral genetics and neuroimaging in the disorder classifications in the *DSM-V*. However, the *Manual *does not explicitly specify what and how neurogenetic, neuroimaging, and neurochemical data can or should be employed in establishing differential diagnoses of mental disorders. Moreover, the possibility of employing these largely experimental neuroscientific and neurotechnological methods for diagnostic purposes is not without contention and has become the focus of considerable neuroethical debate (see [34] for overview).

### Critical deliberations

The history of the *DSM *series may certainly be viewed as an attempt to integrate scientific progress to the categorization of psychopathology, thereby reflecting an increased epistemological capital, and compelling psychiatric diagnoses to be better aligned with the medical model. Yet, while elaboration of a standardized nosology for mental disorders may have afforded a major impetus for research on psychopathology, it has also generated particular problems, abuses and possibly unforeseen consequences in the manner in which psychiatric disturbances are understood, diagnosed, and treated [35].

Perhaps one of the most striking corollaries of the symptom-based, somatically-oriented descriptive approach fostered by the *DSM-III *is the increase in psychopharmacological interventions, applied to conditions ranging from the severest of mental disorders to much milder *DSM *categories that had previously been treated with psychotherapeutic and behavioral approaches. Research in the neurochemistry and pharmacology of specifically defined psychopathological conditions has enabled the pharmaceutical industry to develop drugs targeting biological markers associated with such conditions. While this has led to the relatively successful treatment of a number of neuropsychiatric conditions (e.g., anxiety, depression, certain forms of psychosis) and provided appreciable therapeutic benefit to scores of patients, the pharmacological approach has been less than wholly successful in the treatment of other psychiatric conditions (e.g., type I bipolar disorder, schizophrenia, personality disorders). As well, the potential for misusing the pharmaceutical approach has been decried by several critics [36-39], and the phenomenon of "disease mongering" has been noted in the marketing of various drugs (e.g.- the selective serotonin reuptake inhibitors, SSRIs) for such conditions as "mild social anxiety", which is been described as a "medicalization of shyness" [36]. Similarly, the medicalization of cognition, emotion, and behavior has also generated discourse- if not controversy- about the interpretation of subjective variables, such as what constitutes "normal" or "optimal" function within the context and expectations of society and culture [40].

In this light, the broadening categorization of mental disorders, both in terms of what constitutes "un-health", and who may be a target of psychopharmacological intervention (including young children), has paralleled the increase in the number of individuals considered to possess a mental illness [41]. The perplexing conclusion drawn from a recent National Comorbidity Survey for mental disorders in the United States asserts that: "...about half of Americans will meet the criteria or a *DSM-IV *disorder sometime in their life, with first onset usually in childhood or adolescence. Interventions aimed at prevention or early treatment need to focus on youth" [42], and this prompts a renewed interest in questions of what constitutes treatment, enablement, or enhancement- and what metrics, guidelines, and policies need to be established to clarify such criteria [40,43].

Problematic issues arising in psychiatry, arguably reflecting the large-scale adoption of the *DSM*, may be linked to the difficulties of formulating a standardized nosology of psychopathology. Charles Rosenberg has posited that the formulation of standardized and clearly delineated diagnostic classifications, based on the conceptualization of dysfunctions as discrete disease entities, serves to legitimize existence of named and defined disease(s), and can obscure the 'constructedness' of the categories themselves [44]. It may be worth pondering the extent to which such a phenomenon could be particularly problematic with regard to a number of behavioral and emotional conditions that might be mere extensions of normal behavior or simple "eccentricities" that would then construed as medical diseases. The "pathologization of deviance" and the "medicalization of social ills" are potential effects of psychiatric diagnoses and treatment trends. While such categorizations may arise from, and be directed toward benevolent intentions, caution is required to insure against socio-political usurpation of these diagnoses, and repetition of historical instances of bastardization of medicine by capricious agenda [45-49].

## Conclusion

The history of the *DSM *has been characterized by a shift in the conceptualization of mental health and illness, reflecting an attempt to adhere to the ontological claims and canon of "biomedicine" and sustain psychiatry's medical identity. The evolution of the *DSM *illustrates that what is considered to be "medical" and "scientific" is often not an immutable standard, but rather, may be variable across time and culture, and in this way contingent upon changes in dominant schools of thought. The elaboration of a standardized nosology of mental disorders has had diverse impact(s) on the manner in which psychopathology is conceived, on the definition of who constitutes a psychiatric patient, and how cognitions, emotions, and behaviors are regarded and treated. The act of diagnosis in and of itself validates the very disease it names and defines [44,50]. As such, current classifications of mental disorders must be understood, at least to some extent, as "constructed concepts" that are amenable to modification, as evidenced by the various transmutations within the *DSM *throughout its successive editions, rather than incontestable facts. The development of the *DSM *has evidenced values and assumptions reflective of the *Zeitgeist *of each edition. As noted by John Sadler, "...values, not cognitions, determine what we select as 'important', 'crucial', 'central', 'decisive', or 'related'. In other words, values lend structure to the field of attention, predefining background and foreground, and clustering disparate items into groups. Consequently, descriptive statements about psychopathology issue from presupposed value stances that conceal their own deeper sources, compatibilities, and incompatibilities" [51].

While such values and assumptions may be neither inherently "good" nor "bad", and may be enmeshed within scientific pursuits of all types [52], awareness of their existence and of their contribution to the shaping of what is regarded as scientific knowledge is imperative. Even if we were to presume that scientific knowledge were wholly objective and free of any values or bias whatsoever, it is nonetheless critical to recognize that new information adds to, and may subordinate, older knowledge in an iterative, self-corrective process. Thus, adherence to a doctrinal stance must be flexible to adapt to new insights and revision, lest it become anachronistic and dogmatic. While the changes to the *DSM *are based upon scientific strides and humanitarian intent, it's important to measure such claims by the purported objectives to improve diagnosis and treatment in accordance with both psychiatry's professed medical identity, and the new dimensions of the discipline and its practice that are enabled by neuroscience, neurotechnology, genetics, the social sciences, and the humanities. If, and how such claims are realized by the *DSM-V *remain questions for contemporary users of this new edition - and scholars, researchers and practitioners of psychiatry, the aforementioned fields, and ultimately patients and the public to address and decide. Clearly, both the *DSM *and psychiatry will remain a work-in-progress, and we must be ready and responsible for the potential benefits - and possible problems - that such progress may foster.

## Competing interests

The authors declare that they have no competing interests.
